# *Baat*‐Deficient Mice Recapitulate Elevated 7α‐Hydroxy‐3‐Oxo‐4‐Cholestenoic Acid Observed in a Japanese Patient With *BAAT* Deficiency

**DOI:** 10.1002/jimd.70232

**Published:** 2026-08-05

**Authors:** Soma Koga, Hajime Takei, Ryutaro Tamura, Yugo Takaki, Hiroyuki Kusuhara, Hiroshi Nittono, Hisamitsu Hayashi

**Affiliations:** ^1^ Laboratory of Molecular Pharmacokinetics, Graduate School of Pharmaceutical Sciences, The University of Tokyo Tokyo Japan; ^2^ Junshin Clinic Bile Acid Institute Tokyo Japan; ^3^ Department of Pediatric Gastroenterology and Hepatology, Japanese Red Cross Kumamoto Hospital Kumamoto Japan

**Keywords:** 7α‐hydroxy‐3‐oxo‐4‐cholestenoic acid, adeno‐associated virus, amidation defect, animal models, bile acid metabolism, gene knockdown, genetic disease

## Abstract

Bile acid Coenzyme A: amino acid N‐acyltransferase (*BAAT*) catalyzes the conjugation of bile acids with taurine or glycine, a process essential for bile acid solubility and intestinal lipid absorption. Mutations in *BAAT* cause an inborn error of bile acid metabolism, typically characterized by reduced conjugated bile acids and fat‐soluble vitamin deficiency. However, several clinical features of *BAAT* deficiency cannot be fully explained by impaired conjugation alone, suggesting the presence of broader metabolic disturbances. In this study, we analyzed serum bile acid and intermediate profiles in a Japanese patient with genetically confirmed *BAAT* deficiency and identified a marked elevation of 7α‐hydroxy‐3‐oxo‐4‐cholestenoic acid (7‐HOCA), a key intermediate in bile acid synthesis. To investigate the metabolic consequences of *BAAT* loss, we generated a hepatic *Baat* knockdown mouse model using adeno‐associated virus serotype 8–mediated delivery of Nme2Cas9 and *Baat*‐targeting sgRNA. This model faithfully recapitulated the accumulation of 7‐HOCA observed in the patient, together with a significant reduction in amino acid–conjugated bile acids. Integrated analyses combining bile acid profiling, quantitative PCR, and stable‐isotope tracing of cholesterol demonstrated enhanced cholesterol flux toward bile acid biosynthetic pathways and upregulation of bile acid synthetic enzymes in *Baat*‐deficient livers. These findings indicate that *BAAT* deficiency leads to dysregulated bile acid synthesis in addition to defective conjugation. Together, our results reveal a previously unrecognized metabolic phenotype of *BAAT* deficiency and provide mechanistic insight into its pathophysiology, establishing a novel in vivo model for studying bile acid metabolism beyond simple conjugation defects.

**Abbreviations**
7‐HOCA: 7α‐hydroxy‐3‐oxo‐4‐cholestenoic acid
AAV8: adeno‐associated virus serotype 8
Akr1d1: Δ4‐3‐oxo‐steroid 5β‐reductase
Asbt: apical sodium‐dependent bile acid transporter
BA: bile acid
BAAT: bile acid Coenzyme A: amino acid N‐acyltransferase
C4: 7α‐hydroxy‐4‐cholesten‐3‐one
C4‐26‐ol: 7α, 26‐dihydroxy‐4‐cholesten‐3‐one
CA: cholic acid
C‐BA: conjugated bile acid
CDCA: chenodeoxycholic acid
Cyp27a1: sterol 27‐hydroxylase
Cyp2c70: cytochrome P450, family 2, subfamily c, polypeptide 70
Cyp7a1: cholesterol 7α‐hydroxylase
Cyp7b1: oxysterol 7α‐hydroxylase
Cyp8b1: sterol 12α‐hydroxylase
D‐Bil: direct bilirubin
DCA: deoxycholic acid
Fgf15: fibroblast growth factor 15
Gapdh: glyceraldehyde‐3‐phosphate dehydrogenase
GCA: glycocholic acid
GCDCA: glycochenodeoxycholic acid
GUDCA: glycoursodeoxycholic acid
GUDCA‐3S: glycoursodeoxycholic acid‐3‐sulfate
Hsd3b7: 3β‐hydroxy Δ5‐C27‐steroid dehydrogenase/isomerase
Ibabp: ileal bile acid‐binding protein
KD: knockdown
KO: knockout
LC–MS/MS: liquid chromatography with tandem mass spectrometry system
ND: not detected
Osta: organic anion transporter α
Ostb: organic anion transporter β
sgRNA: small‐guide RNA
Slc27a5: solute carrier family 27 member 5
T‐Bil: total bilirubin
TCA: taurocholic acid
TCDCA: taurochenodeoxycholic acid
TUDCA: tauroursodeoxycholic acid
TUDCA‐3S: tauroursodeoxycholic acid‐3‐sulfate
T‐α‐MCA: tauro‐α‐muricholic acid
T‐β‐MCA: tauro‐β‐muricholic acid
T‐ω‐MCA: tauro‐ω‐muricholic acid
U‐BA: unconjugated bile acid
UDCA: ursodeoxycholic acid
α‐MCA: α‐muricholic acid
β‐MCA: β‐muricholic acid
ω‐MCA: ω‐muricholic acid

## 1 Introduction

Bile acids (BAs) are amphipathic sterol derivatives synthesized from cholesterol, an insoluble lipid component of biological membranes [1]. They are produced in the liver, secreted into bile, and subsequently released into the small intestine, where they facilitate the absorption of hydrophobic nutrients, including dietary lipids and fat‐soluble vitamins [2]. BAs are synthesized mainly through two pathways: the classical (neutral) pathway, initiated by cholesterol 7α‐hydroxylase (*CYP7A1*), which generates cholic acid (CA) and chenodeoxycholic acid (CDCA); and the alternative (acidic) pathway, initiated by sterol 27‐hydroxylase (*CYP27A1*), which predominantly yields CDCA in humans. In humans, CA and CDCA are the major primary BAs. In mice, however, the primary BA pool differs substantially and consists predominantly of CA and muricholic acids (MCAs), particularly α‐ and β‐MCA, with CDCA being further metabolized to MCAs [3, 4, 5]. These multi‐enzymatic reactions involve modifications of the steroid nucleus, oxidation of the side‐chain, and amino acid conjugation. The final step, amino acid conjugation, is catalyzed by bile acid coenzyme A: amino acid N‐acyltransferase (*BAAT*) [2]. Conjugation with glycine or taurine markedly increases the water solubility of BAs and reduces their toxicity [6]. Notably, glycine‐conjugated BAs are major BA species in humans, whereas taurine‐conjugated BAs predominate in mice [4].

*BAAT* deficiency, an inborn error of BA synthesis [7, 8, 9], is characterized by markedly reduced taurine‐ and glycine‐conjugated BAs, fat‐soluble vitamin deficiency, neonatal cholestasis, and growth failure [10, 11, 12, 13]. Because amino acid conjugation enhances the amphipathic nature of BAs, its loss impairs micelle formation and intestinal absorption of dietary lipids and fat‐soluble vitamins [6, 13]. These features account for the typical clinical manifestations of *BAAT* deficiency. However, several patients also present with hepatocellular injury, cholestasis, or cholangiopathic changes that cannot be fully explained by conjugation failure alone, suggesting broader disturbances in BA metabolism [13]. Although mouse models of *BAAT* deficiency reproduce the conjugation defect and associated fat‐soluble vitamin malabsorption [14, 15], the metabolic consequences beyond amino acid conjugation defects have not been systematically characterized. Clarifying these alterations may therefore provide new insights into the pathophysiology of *BAAT* deficiency, which likely involves a complex reprogramming of BA metabolism rather than a mere loss of conjugation activity.

Given these considerations, in this study, we investigated the metabolic consequences of *BAAT* deficiency with a particular focus on BA biosynthesis and its intermediates. We first analyzed the serum of a Japanese patient with genetically confirmed *BAAT* deficiency and identified a marked elevation of 7α‐hydroxy‐3‐oxo‐4‐cholestenoic acid (7‐HOCA), a key intermediate in BA synthesis. To further explore this metabolic alteration, we established a liver‐directed *Baat* knockdown (KD) mouse model using adeno‐associated virus serotype 8 (AAV8) vector‐mediated delivery of Nme2Cas9 and *Baat*‐targeting sgRNA. By combining biochemical assays, quantitative PCR (qPCR), BA profiling, and stable isotope–tracing analyses, we sought to clarify how *BAAT* deficiency alters the bile acid metabolic network and leads to 7‐HOCA accumulation. This study provides mechanistic insight into the interplay between BA conjugation and biosynthesis, revealing an unrecognized link between *BAAT* function and cholesterol catabolism. The *Baat* KD mouse model established here serves as a valuable tool for investigating abnormal BA metabolism beyond amino acid conjugation defects and for advancing our understanding of *BAAT* deficiency.

## 2 Methods

The reagents used in this study were of analytical grade and are listed in Table S1.

### 2.1 Human Specimens

The study involving human subjects was approved by the institutional review board of the Graduate School and Faculty of Medicine, Kyoto University (approval number: G1253). This study was conducted as part of the nationwide registry study CIRCLe (Comprehensive and Informative Registry system for Childhood Liver disease), in which genetic and bile acid analyses are routinely performed for all enrolled patients. The study adhered to the principles of the Declaration of Helsinki (1964) and its subsequent amendments, or to comparable ethical standards. Informed consent was obtained primarily through electronic signatures via a secure online system approved by the ethics committees, and in some cases, written consent was obtained. Consent was obtained from all participants or from their parents/guardians for minors under 18 years of age.

Among the patients registered in CIRCLe, a Japanese male with biallelic mutations in the *BAAT* gene was identified and clinically characterized in detail. This patient had been previously reported [12]. Using targeted gene panel sequencing with a next‐generation sequencer, he was diagnosed with *BAAT* deficiency, being compound heterozygous for NM_001701.4:c.58C>T; p.(Arg20Ter) and NM_001701.4:c.953G>A; p.(Gly318Glu). The patient received oral ursodeoxycholic acid (UDCA) therapy during two separate periods: from 1 to 4 months of age, and from 3 years and 10 months of age to 6 years and 4 months of age. Age‐matched non‐cholestatic liver disease patients (*n* = 4) and cholestatic patients (*n* = 5) served as the comparison groups. Clinical parameters for all subjects are summarized in Table 1.

**TABLE 1:** Biochemical test results in a patient with *BAAT* deficiency and in non‐cholestatic and cholestatic patients.

Group	UDCA status	Age	AST (U/L)	ALT (U/L)	GGT (U/L)	T‐Bil (mg/dL)	D‐Bil (mg/dL)	
*BAAT* deficiency	on UDCA	5y5m	32	11	7	0.7	0.04	
	off UDCA	6y7m	33	13	9	0.9	0.06	
Non‐cholestatic (*n* = 4)	off UDCA	4y9m (3y2m–6y2m)	37 (29–82)	23 (10–132)	19.5 (11–33)	0.45 (0.3–1)	0.1 (0.1–0.3)	
Cholestatic (*n* = 5)	on UDCA	3y3m (2y4m–7y11m)	218 (63–738)	198 (34–785)	71 (26–541)	6.5 (4.4–21)	4.9 (2.1–13.9)	

*Note:* Values for the BAAT‐deficient patient were obtained during UDCA therapy (age 5y5m) and outside the UDCA‐treatment period (age 6y7m). Values for non‐cholestatic patients (*n* = 4) and cholestatic patients (*n* = 5) are presented as median (range).

Abbreviations: ALT, alanine aminotransferase; AST, aspartate aminotransferase; D‐Bil, direct bilirubin; GGT, γ‐glutamyl transferase; T‐Bil, total bilirubin; UDCA, ursodeoxycholic acid.

### 2.2 Animals and Diets

All mouse experiments were approved by the animal experiment committee of the University of Tokyo (permission number: A2024P003–01) and performed in accordance with the institutional guidelines and the ARRIVE guidelines. Male C57BL/6JJcl mice were purchased from CLEA Japan Inc. (Tokyo, Japan) for each experimental series. Upon arrival, mice were acclimated in the animal facility of the University of Tokyo before the start of experiments. Mice were housed in plastic cages under conventional housing conditions with controlled temperature (23.5°C ± 2.5°C), humidity (52.5% ± 12.5%), and a 12‐h light/dark cycle, and had free access to water and a standard chow diet (CE‐2; CLEA Japan Inc., Tokyo, Japan).

### 2.3 SgRNA Design and Plasmid Construction

Single‐guide RNAs (sgRNAs) targeting the *Baat* gene were designed using Chop‐Chop (https://chopchop.cbu.uib.no/), and two candidate sequences were selected. The selected oligonucleotides were synthesized with the following modifications: forward primer, 5′‐ACCG‐[Selected sequence]‐3′; reverse primer, 5′‐AAC‐[Complementary sequence of the selected sequence]‐C‐3′ (Eurofins Genomics K.K., Tokyo, Japan). The sgRNA sequences used in this study are listed in Table S2. Each pair of complementary oligonucleotides was annealed and cloned into the Nme2Cas9_AAV, which was a gift from Erik Sontheimer (#119924; Addgene, Watertown, MA) [16].

### 2.4 AAV Vector Production and Purification

AAV vectors were produced and purified as described previously [17].

### 2.5 Animal Studies

Male C57BL/6JJcl mice (8 weeks old) were intraperitoneally injected during the light phase with AAV8 vectors carrying Nme2Cas9 and either *Baat*‐targeting sgRNA or a corresponding control sgRNA at a total dose of 1 × 10^12^ genome copies. Mice were purchased separately, and each experimental group was housed in a separate cage after random allocation. Two weeks after AAV8 vector administration, the mice were anesthetized with isoflurane (2%, inhalation anesthesia apparatus), and a midline laparotomy was performed in the procedure room. The livers were harvested, snap‐frozen in liquid nitrogen, and stored at −80°C for biochemical analyses or processed for quantitative PCR (qPCR). Blood was collected from the inferior vena cava into heparinized tubes, and plasma was separated by centrifugation at 1700 × g for 15 min at 4°C, snap‐frozen in liquid nitrogen, and stored at −80°C. Bile was aspirated from the gallbladder, snap‐frozen in liquid nitrogen, and stored at −80°C. Primary hepatocytes were isolated according to a previous protocol [18] with minor modifications to the plating medium composition, which is summarized in Table S3.

The small intestine was collected and processed as follows. For BA analysis, the small intestine was excised and rinsed with ice‐cold PBS and PBS containing 0.5% BSA. Terminal ileum was then collected and analyzed as described in the BA analysis section. For qPCR, the small intestine was excised and rinsed with ice‐cold PBS containing 0.5 mM taurocholic acid. The distal half of the rinsed tissue was opened longitudinally, cut into small fragments, and incubated in 20 mM EDTA/PBS for 60 min at 4°C. After centrifugation at 20 × g, the supernatant was discarded. The pellet was then incubated again with 20 mM EDTA/PBS for 30 min at 4°C. After centrifugation at 20 × g, the supernatants was collected. The remaining pellet was incubated once more with 20 mM EDTA/PBS for 30 min at 4°C, followed by centrifugation at 20 × g. The supernatants obtained from second and third EDTA incubations were pooled and centrifuged at 300 × g for 5 min at 4°C. The resulting pellet was then resuspended in TrypLE express (Thermo Fisher Scientific) and incubated at 37°C for 1 min, followed by filtration through a 40‐μm cell strainer to obtain intestinal epithelial cells. The collected cells were subsequently centrifuged at 1000 × g for 5 min at 4°C and used for RNA extraction.

For data analysis, only mice whose liver *Baat* mRNA expression level (normalized to glyceraldehyde‐3‐phosphate dehydrogenase (*Gapdh*)) was less than one‐fifth of the mean value of the control group were included.

### 2.6 Assessment of ^13^C_5_ ‐Cholesterol Metabolism

Primary hepatocytes (4.0 × 10^5^ viable cells) were incubated with 10 μg/mL of ^13^C_5_‐cholesterol (Santa Cruz Biotechnology Inc., Dallas, TX) at 37°C with end‐over‐end rotation for 15, 30, or 60 min. The reactions were quenched by placing the samples on ice and then centrifuged at 200 × g for 3 min at 4°C. The supernatants were removed, and cell pellets were snap‐frozen in liquid nitrogen, and stored at −80°C until BA analysis.

### 2.7 BA Analysis

BA concentrations and profiles in human serum and mouse liver, plasma, gallbladder bile, and primary hepatocytes were analyzed using a liquid chromatography–tandem mass spectrometry (LC–MS/MS) system [19, 20]. Total BA levels in terminal ileal tissue were measured using a Total Bile Acid‐Test Wako Kit (FUJIFILM Wako Pure Chemical, Osaka, Japan), as described below.

Human serum samples were analyzed as described previously [19, 20]. For mouse studies, liver samples (20 mg) were processed as described previously [19], and 0.5 mL aliquots were analyzed. Plasma samples (20 μL) were processed in the same manner, with 0.1 mL aliquots subjected to analysis. Gallbladder bile samples (1 μL) were diluted 1000‐fold with distilled water and purified using a solid‐phase extraction cartridge as described in the previous report [20]. The eluates were evaporated to dryness, reconstituted in 50% ethanol, and then analyzed by LC–MS/MS.

For ^13^C_5_‐cholesterol metabolite analysis, the cell pellets were mixed with 50 μL of acetonitrile and vortexed for 30 s. After centrifugation, the resulting supernatant was collected, evaporated to dryness, reconstituted in 80% methanol, and analyzed [21]. Unlabeled standards were used for quantification of the ^13^C_5_‐labeled metabolites.

For measurement of total BA levels in the ileum, terminal ileal tissue (50 mg) was homogenized in 200 μL of methanol/acetonitrile (1:1, v/v) using a Tissue Lyser II (QIAGEN, Hilden, Germany). The homogenates were sonicated at the high setting with repeated cycles of 10 s on and 20 s off for 5 min, and then centrifuged at 3000 × g for 5 min at 4°C. The supernatants were collected, and half of each supernatant was concentrated using a centrifugal concentrator for 30 min. The residues were resuspended in 100 μL of 80% ethanol, and total BA levels were measured using a Total Bile Acid‐Test Wako Kit according to the manufacturer's instructions.

### 2.8 Quantitative PCR (qPCR)

QPCR was performed with minor modifications to the protocol described previously [17]. *Gapdh* expression was used as an internal control for normalization. The primer sequences used in this study are listed in Table S4.

### 2.9 Statistical Analysis

All graphs are presented as mean ± SEM. Differences between the two groups were assessed using Welch's *t*‐test at a 95% confidence level. Statistical analyses were performed using GraphPad Prism 9.5.1 (GraphPad Software, La Jolla, CA).

### 2.10 Use of AI Tools

An AI‐based large language model (ChatGPT, OpenAI, San Francisco, CA) was used solely for English language editing and improvement of grammar and clarity during manuscript preparation. The AI tool was not used for data analysis, interpretation, figure generation, or generation of scientific conclusions.

## 3 Results

### 3.1 Increase in 7‐HOCA in a *BAAT* ‐Deficient Patient

**FIGURE 1:**
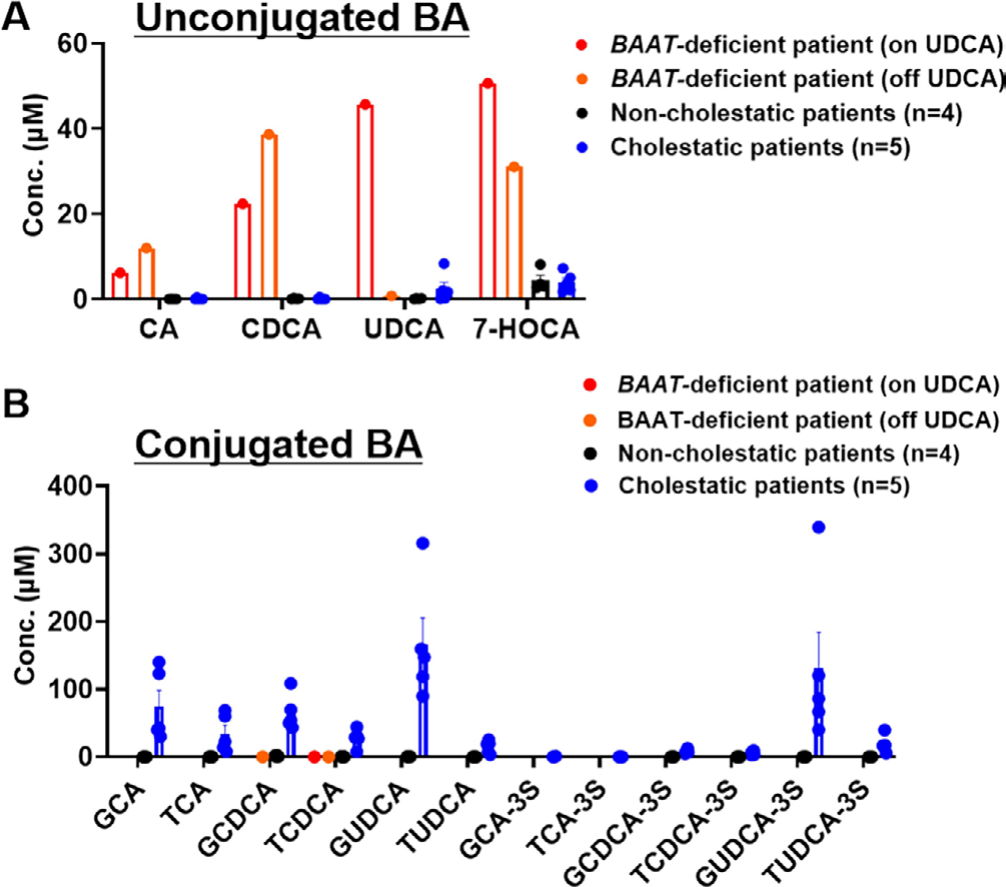
Serum BA profiles of the *BAAT‐*deficient patient and non‐cholestatic or cholestatic patients. Serum unconjugated (A) and conjugated (B) BA levels were quantified by LC–MS/MS. Values for the *BAAT‐*deficient patient are shown for samples obtained both during and outside UDCA therapy. Values for the non‐cholestatic and cholestatic comparison patients include samples obtained with and without UDCA administration. Some values were below the detection limit and thus are not shown in the plots. 7‐HOCA, 7α‐hydroxy‐3‐oxo‐4‐cholestenoic acid; BA, bile acid; CA, cholic acid; CDCA, chenodeoxycholic acid; DCA, deoxycholic acid; GCA, glycocholic acid; GCA‐3S, glycocholic acid 3‐sulfate; GCDCA, glycochenodeoxycholic acid; GCDCA‐3S, glycochenodeoxycholic acid 3‐sulfate; GUDCA, glycoursodeoxycholic acid; GUDCA‐3S, glycoursodeoxycholic acid 3‐sulfate; TCA, taurocholic acid; TCA‐3S, taurocholic acid 3‐sulfate; TCDCA, taurochenodeoxycholic acid; TCDCA‐3S, taurochenodeoxycholic acid 3‐sulfate; TUDCA, tauroursodeoxycholic acid; TUDCA‐3S, tauroursodeoxycholic acid 3‐sulfate; UDCA, ursodeoxycholic acid.

Clinical and biochemical characteristics of a Japanese male patient with *BAAT*‐deficiency and age‐matched non‐cholestatic liver disease patients (*n* = 4) and cholestatic patients (*n* = 5) are summarized in Table 1. Serum bile acid profiles were compared between a Japanese male patient with *BAAT*‐deficiency and age‐matched cholestatic patients (*n* = 5) (Figure 1). Serum 7‐HOCA levels were further compared with those in non‐cholestatic patients and cholestatic patients (Table 2). In the *BAAT*‐deficient patient, both taurine‐ and glycine‐conjugated BA were nearly absent, consistent with defective bile acid conjugation. UDCA was detectable only during UDCA therapy. Notably, a substantial amount of 7‐HOCA was present in the serum of the *BAAT*‐deficient patient both during and outside the periods of UDCA administration (Figure 1 and Table 2). In contrast, 7‐HOCA was undetectable in the patient's urine (data not shown).

**TABLE 2:** Serum 7‐HOCA levels in a patient with BAAT deficiency and in non‐cholestatic and cholestatic patients.

	*BAAT* deficiency		Non‐cholestatic (*n* = 4)	Cholestatic (*n* = 5)
	on UDCA	off UDCA		
7‐HOCA (μM)	50.67	31.08	3.29 (2.69–8.13)	3.60 (1.75–7.27)

*Note:* Values for the *BAAT*‐deficient patient were obtained during UDCA therapy and outside the UDCA‐treatment period. Values for non‐cholestatic liver disease patients (*n* = 4) and cholestatic patients (*n* = 5) are presented as median (range).

Abbreviation: 7‐HOCA, 7α‐hydroxy‐3‐oxo‐4‐cholestenoic acid.

### 3.2 Generation of *Baat* KD Mice

**FIGURE 2:**
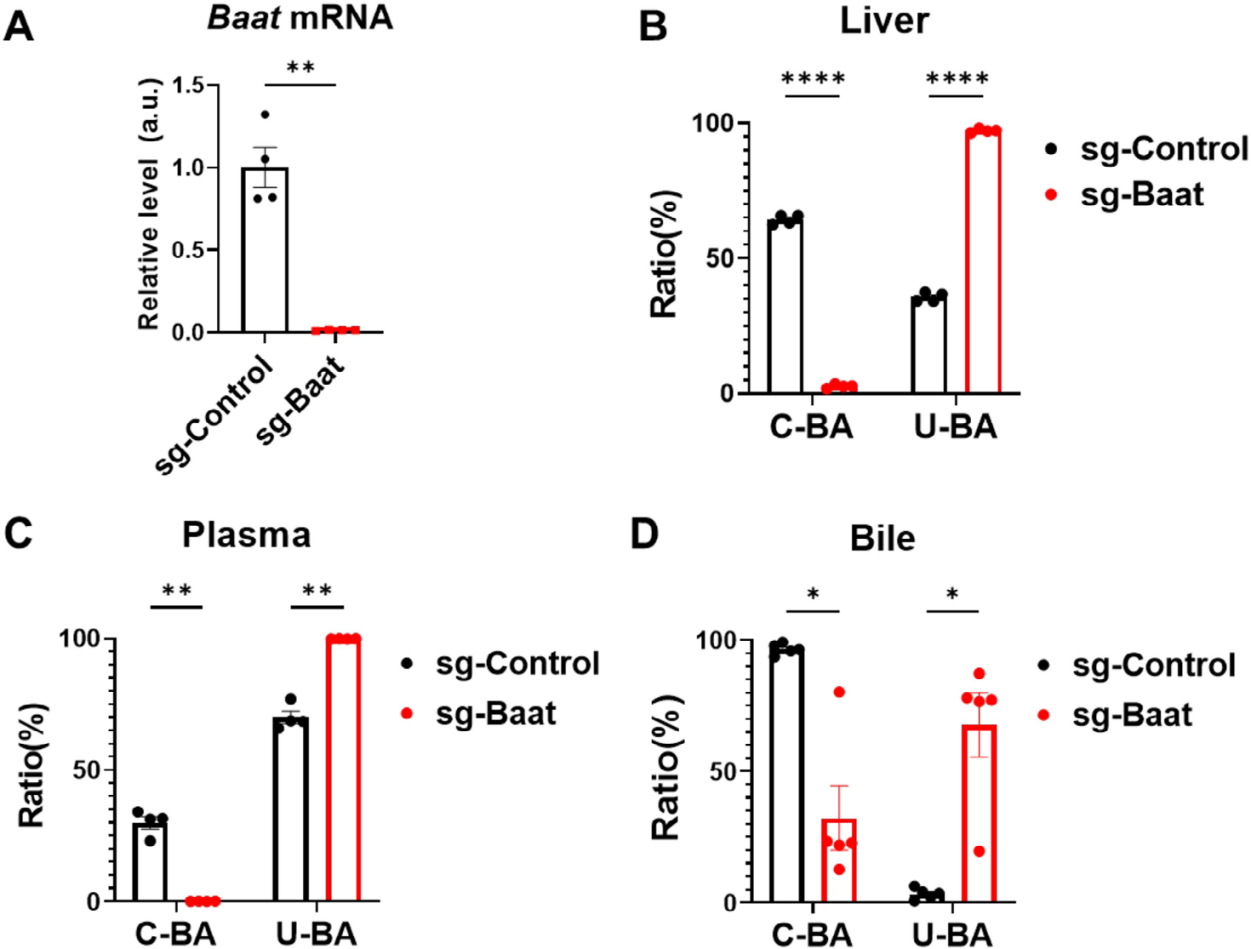
Generation of *Baat* KD mice. Male mice (8 weeks old, *n* = 4–5 per group) were administered AAV8 vectors expressing sgRNA targeting *Baat* (sg‐Baat) or a control sgRNA (sg‐Control). The mice were sacrificed at 10 weeks of age, and the liver, plasma, and bile were collected. (A) *Baat* mRNA expression in the liver was quantified by qPCR and normalized to *Gapdh* mRNA expression. (B–D) BAs and intermediates in the liver (B), plasma (C), and bile (D) were quantified by LC–MS/MS. Conjugated and unconjugated bile acids are shown as their ratio to the sum of all quantified compounds. All data are expressed as mean ± SEM. **p < 0.01, ****p < 0.0001 by Welch's *t*‐test. C‐BA, conjugated bile acid; U‐BA, unconjugated bile acid.

**FIGURE 3:**
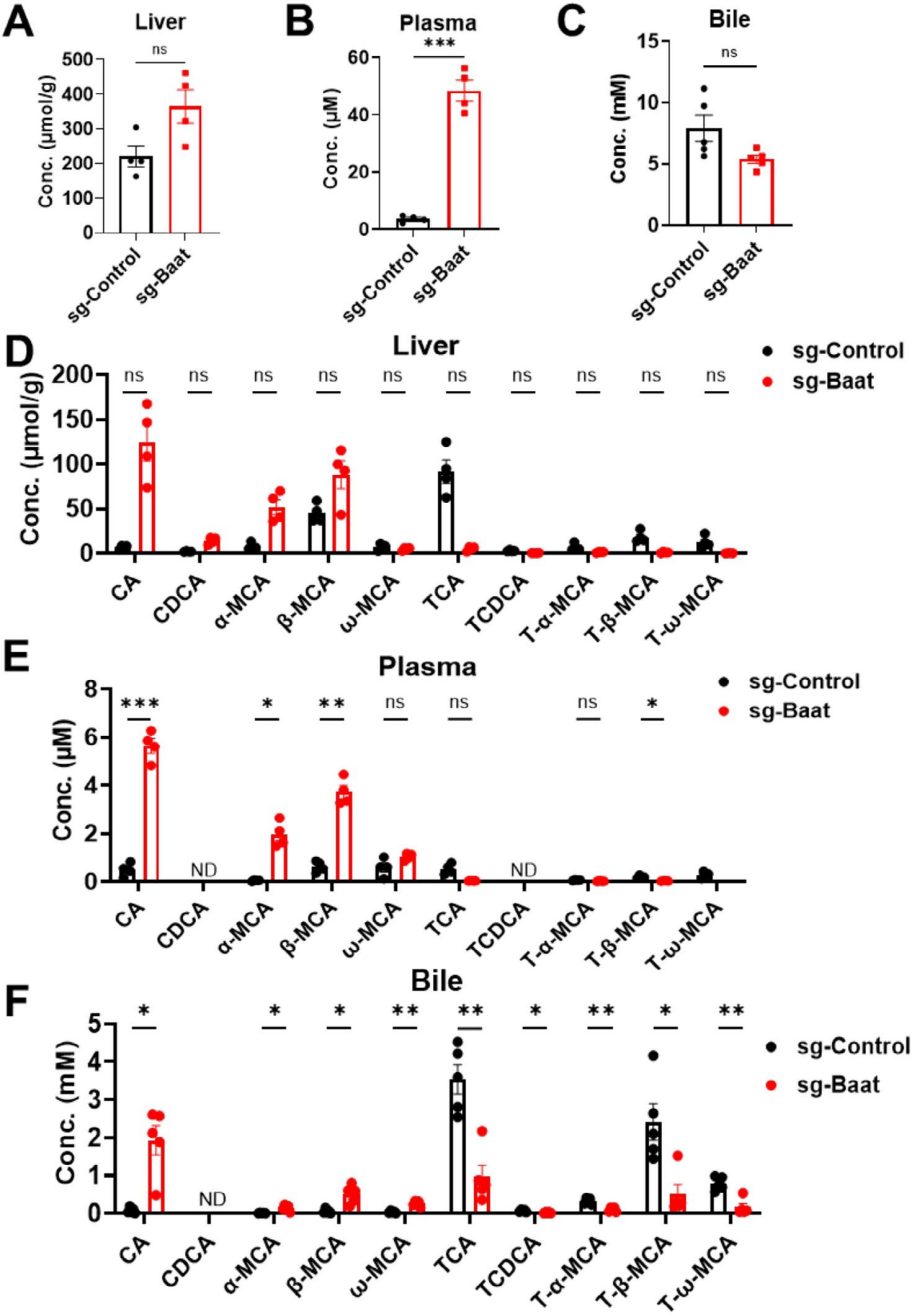
BA profiles in *Baat* KD mice. *Baat* KD mice were generated as described in Figure 2, and liver, plasma, and bile were collected 2 weeks after AAV8‐sgRNA administration. BAs and intermediates in these specimens were quantified by LC–MS/MS. (A–C) Concentrations of all detected species in the liver (A), plasma (B), and bile (C). (D–F) Concentrations of individual BAs and their taurine‐conjugated forms in the liver (D), plasma (E), and bile (F). Some values were below the detection limit and are not shown in the plots. All data are presented as mean ± SEM. *p < 0.05, **p < 0.01, ***p < 0.001 by Welch's *t*‐test. BA, bile acid; CA, cholic acid; CDCA, chenodeoxycholic acid; DCA, deoxycholic acid; ND, not detected; TCA, taurocholic acid; TCDCA, taurochenodeoxycholic acid; TDCA, taurodeoxycholic acid; T‐α‐MCA, tauro‐α‐muricholic acid; T‐β‐MCA, tauro‐β‐muricholic acid; T‐ω‐MCA, tauro‐ω‐muricholic acid; α‐MCA, α‐muricholic acid; β‐MCA, β‐muricholic acid; ω‐MCA, ω‐muricholic acid.

To investigate the mechanism underlying the increase in 7‐HOCA, we generated a murine model of *Baat* deficiency via liver‐directed *Baat* knockdown (KD). This was achieved by hepatotropic AAV8‐mediated co‐delivery of *Baat*‐targeting sgRNA and Nme2Cas9 to 8‐week‐old male mice. Two weeks after AAV8 administration, the mice were analyzed. Hepatic *Baat* mRNA expression was markedly reduced in *Baat* KD mice compared with control mice (Figure 2A), which was functionally supported by a pronounced decrease in the ratio of taurine‐conjugated to unconjugated BA in the liver, plasma, and bile (Figure 2B–D). Glycine‐conjugated species were either undetectable or present only at trace levels in both groups. The total concentrations of BAs and their intermediates were increased in the liver and plasma, but decreased in the bile of *Baat* KD mice compared with controls (Figure 3A–C). In both *Baat* KD and control mice, the major BA species were cholic acid, α‐MCA, and β‐MCA, which were predominantly present in unconjugated form in *Baat* KD mice and in taurine‐conjugated form in controls (Figure 3D–F).

### 3.3 Increase in 7‐HOCA in *Baat* KD Mice

**FIGURE 4:**
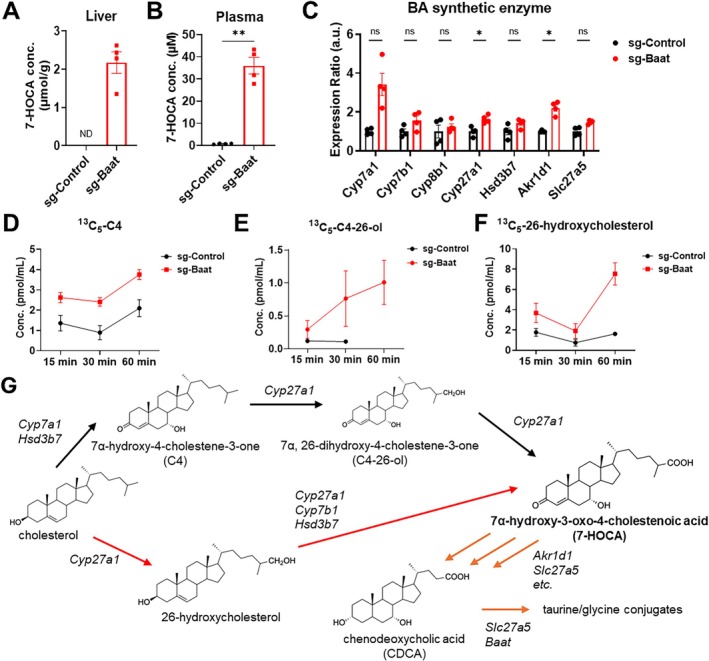
Increased 7‐HOCA levels in *Baat* KD mice. *Baat* KD mice were generated as described in Figure 2, and liver, plasma, and bile were collected 2 weeks after AAV8‐sgRNA administration. BAs and intermediates in these specimens were quantified by LC–MS/MS. (A, B) Concentrations of 7α‐hydroxy‐3‐oxo‐4‐cholestenoic acid (7‐HOCA) in the liver (A) and plasma (B). (C) Liver expression levels of genes involved in BA synthesis were quantified by qPCR and normalized to *Gapdh* mRNA. (D–F) Assessment of BA biosynthetic function. Primary hepatocytes were isolated from mice transduced with AAV‐sgRNA (*n* = 3 per group), incubated with ^13^C_5_‐cholesterol, and analyzed by LC–MS/MS. Levels of ^13^C_5_‐7α‐hydroxy‐4‐cholestene‐3‐one (^13^C_5_‐C4) (D), ^13^C_5_‐7α, 26‐dihydroxy‐4‐cholestene‐3‐one (^13^C_5_‐C4‐26‐ol) (E), and ^13^C_5_‐26‐hydroxycholesterol (F) are shown. Some values were below the detection limit and are not shown in the plots. All data are presented as mean ± SEM. *p < 0.05, **p < 0.01 by Welch's *t*‐test. (G) BA metabolism pathway. Black and red arrows indicate the classical (neutral) and alternative (acidic) pathways, respectively. 7‐HOCA, 7α‐hydroxy‐3‐oxo‐4‐cholestenoic acid; *Akr1d1*, Δ4‐3‐oxo‐steroid 5β‐reductase; *Cyp27a1*, sterol 27‐hydroxylase; *Cyp7a1*, cholesterol 7α‐hydroxylase; *Cyp7b1*, oxysterol 7α‐hydroxylase; *Cyp8b1*, sterol 12α‐hydroxylase; *Hsd3b7*, 3β‐hydroxy Δ5‐C27‐steroid dehydrogenase/isomerase; ND, not detected; *Slc27a5*, solute carrier family 27 member 5.

To determine whether the increase in 7‐HOCA observed in the *BAAT*‐deficient patient was recapitulated in the *Baat* KD mice, 7‐HOCA levels were quantified in multiple tissues. In the liver, 7‐HOCA was detected exclusively in *Baat* KD mice (Figure 4A). In plasma, it was measurable in both groups but significantly higher in the *Baat* KD group (Figure 4B). In contrast, 7‐HOCA was undetectable in bile or urine in either group (data not shown). These findings closely mirrored the pattern observed in the *BAAT*‐deficient patient (Figure 1).

### 3.4 Alterations in the BA Biosynthesis

To further characterize metabolic changes associated with *Baat* KD, the expression of genes involved in BA biosynthesis (*Cyp7a1*, *Cyp7b1*, *Cyp8b1*, *Cy*p*27a1*, *Hsd3b7*, *Akr1d1*, and *Slc27a5*) was quantified (Figure 4C). *Baat* KD mice exhibited an overall upregulation trend, with significant increases in *Cyp27a1* and *Akr1d1* expression. Upregulation of the initial cholesterol‐catabolizing enzymes *Cyp7a1* and *Cyp27a1* [2] may account for the expanded BA pool observed in *Baat* KD mice (Figure 3A,B). To directly assess hepatic BA biosynthesis activity, ^13^C_5_‐cholesterol metabolism was examined in primary hepatocytes (Figure 4D–F). After incubation with ^13^C_5_‐cholesterol, levels of the intermediates 7α‐hydroxy‐4‐cholesten‐3‐one (^13^C_5_‐C4), 7α, 26‐dihydroxy‐4‐cholesten‐3‐one (^13^C_5_‐C4‐26‐ol), and ^13^C_5_‐26‐hydroxycholesterol increased over time and were generally higher in hepatocytes derived from *Baat* KD mice. Some samples exhibited concentrations below the detection limit for ^13^C_5_‐C4‐26‐ol and ^13^C_5_‐26‐hydroxycholesterol (Figure 4E,F).

### 3.5 Alterations in Ileal BA Levels and FGF15 Signaling

**FIGURE 5:**
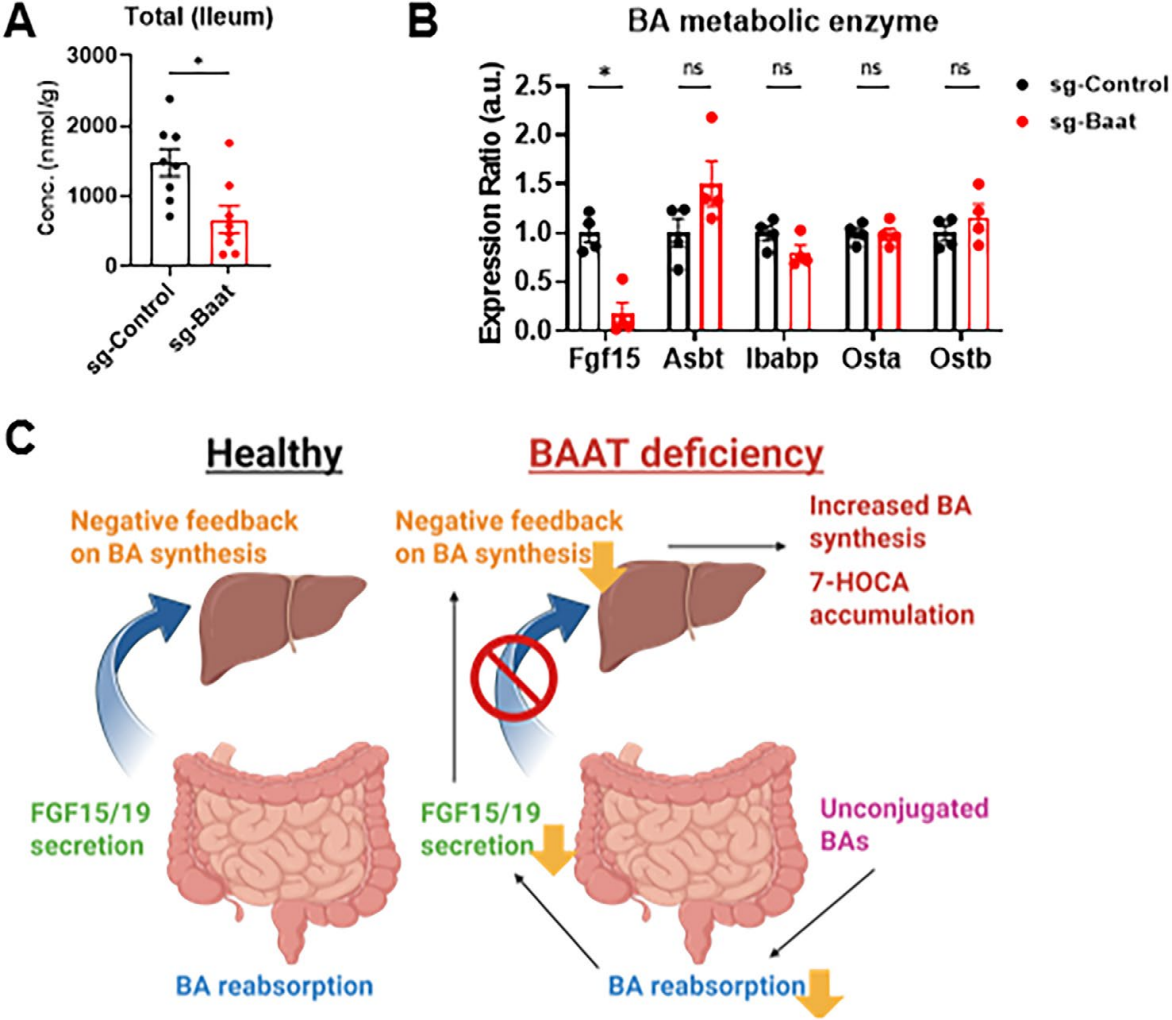
Reduced ileal BA levels and *Fgf15* expression in *Baat* KD mice. *Baat* KD mice were generated as described in Figure 2, and ileal tissues (A) or intestinal epithelial cells (B) were collected 2 weeks after AAV8‐sgRNA administration. (A) Total ileal BA levels. (B) mRNA expression levels of *Fgf15* and genes involved in ileal BA transport, normalized to *Gapdh* mRNA expression. All data are presented as mean ± SEM. *p < 0.05 by Welch's *t*‐test. (C) Proposed mechanism by which reduced BA conjugation may decrease ileal BA exposure, attenuate FGF15‐mediated feedback, and enhance hepatic cholesterol flux toward BA synthesis. Created in BioRender. Koga, S. (2026) https://BioRender.com/ccim9ll. Asbt, apical sodium‐dependent bile acid transporter; Fgf15, fibroblast growth factor; Ibabp, ileal bile acid‐binding protein; Osta, organic anion transporter; α; Ostb, organic anion transporter β.

To gain further insights into the regulation of BA dynamics, total BA levels and the expression of genes involved in ileal BA transport and FXR–FGF15 signaling (*Fgf15*, *Slc10a2*, *Fabp6*, *Slc51a*, and *Slc51b*) were evaluated in the distal intestine (Figure 5A,B). *Baat* KD mice showed a significant reduction in total ileum BA levels, supporting decreased ileal BA exposure and/or reduced intestinal BA reabsorption (Figure 5A). Ileal *Fgf15* expression was also significantly decreased, whereas the expression of BA transport‐related genes, including *Slc10a2, Fabp6, Slc51a, and Slc51b*, was not significantly altered (Figure 5B). These findings suggest that reduced ileal BA exposure, rather than transcriptional downregulation of intestinal BA transporters, contributes to attenuation of the ileal FGF15 feedback pathway in *Baat* KD mice.

## 4 Discussion

This study identifies an unexpected metabolic alteration in *BAAT* deficiency, revealing that 7‐HOCA, a BA synthesis intermediate, accumulates in both a *BAAT*‐deficient patient and a liver‐directed *Baat* KD mouse model (Figures 1 and 4A,B, and Table 2). Although *BAAT* deficiency is classically recognized as a terminal defect in amino acid conjugation, our results indicate broader remodeling of cholesterol and BA metabolism and provide an in vivo platform for investigating BA homeostasis.

In the *BAAT*‐deficient patient, conjugated BAs were nearly absent, consistent with a loss of conjugation activity, while unconjugated species and 7‐HOCA were elevated (Figure 1 and Table 1). Notably, 7‐HOCA remained detectable during and outside UDCA therapy, indicating that BA supplementation did not normalize this intermediate. The same metabolic phenotype was reproduced in *Baat* KD mice (Figure 4A,B), supporting the view that *BAAT* deficiency perturbs upstream BA synthesis.

Mechanistically, the increased hepatic expression of *Cyp27a1* and *Akr1d1* in *Baat* KD mice (Figure 4C) suggests a compensatory upregulation of metabolic enzymes within the cholesterol–BA metabolic axis. Rather than a reduction in the total BA pool size, our data indicate a redistribution with increased hepatic and plasma BA and decreased biliary BA concentrations (Figure 3A–C), consistent with impaired biliary export and altered enterohepatic flux. Importantly, our *Baat* KD mice exhibited significantly reduced total BA levels in the ileum together with decreased ileal *Fgf15* expression (Figure 5A,B). A similar pattern was previously reported in *Baat* KO mice [14]. Unconjugated BAs are poorer substrates for ASBT than their conjugated counterparts [22], resulting in less efficient absorption in the terminal ileum, where farnesoid X receptor (FXR) is highly expressed [23]. These findings support a model in which reduced BA conjugation decreases ileal BA exposure, thereby attenuating intestinal FXR–FGF15 feedback. Because FGF15 suppresses hepatic *Cyp7a1* expression [24, 25, 26], reduced FGF15 signaling may lead to derepression of hepatic BA synthesis and enhanced cholesterol flux toward BA biosynthesis (Figure 5C). A similar pattern has been described in patients treated with cholestyramine or after ileal resection, where *CYP7A1* activity and plasma 7‐HOCA increase together and are accompanied by higher C4, consistent with activation of the classical pathway initiated by *CYP7A1* [27, 28, 29, 30]. Consistent with this model, our *Baat* KD mice showed elevated levels of C4 and its downstream intermediate C4‐26‐ol [29] together with an overall upregulation trend in BA biosynthesis genes (Figure 4C–E), supporting activation of the classical pathway.

In addition to activation of the classical pathway, our data also suggest a potential role for the alternative (acidic) pathway in 7‐HOCA accumulation. In this pathway, 7‐HOCA is generated through the sequential actions of *CYP27A1*, *CYP7B1*, and *HSD3B7* [29, 31, 32] and subsequently processed toward primary BAs by *AKR1D1* and downstream enzymes [32]. In line with previous observations in *Baat* KO mice [14], *Cyp27a1* expression was significantly elevated in our *Baat* KD mice, consistent with increased levels of 26‐hydroxycholesterol and suggesting enhanced flux through the acidic branch (Figure 4C,F). Conversely, *Akr1d1* was also upregulated, which would be expected to facilitate 7‐HOCA metabolism rather than its accumulation. This discrepancy may indicate post‐transcriptional regulation or differences in enzyme activity. Direct activity assays will be necessary to define the relative contributions of each pathway to 7‐HOCA elevation in BAAT deficiency.

The observation that 7‐HOCA accumulated in serum but not in bile or urine suggests restricted canalicular excretion and limited renal clearance, potentially increasing systemic exposure (Figure 4A,B). Given its structural similarity to oxysterols, persistently elevated 7‐HOCA may carry signaling implications with potential effects on lipid and BA homeostasis [33, 34]. Moreover, the increase of total BAs and intermediates in the liver and plasma of *Baat* KD mice, together with their reduction in bile, is compatible with impaired BA export and altered enterohepatic circulation (Figure 3A–C). While overt liver injury was not evident in these mice, such metabolic alterations could represent an early stage of cholestatic stress. This interpretation is consistent with clinical observations in some *BAAT*‐deficient patients [10, 13], who exhibit mild to moderate cholestasis and hepatocellular injury not fully explained by fat‐soluble vitamin deficiency. The lack of hepatotoxicity in our model likely reflects species differences in BA composition: in mice, the BA pool is dominated by muricholic acids, which are more hydrophilic and less cytotoxic than the cholic and chenodeoxycholic acids prevalent in humans [4]. This hydrophilic composition, maintained by the murine‐specific enzyme *Cyp2c70*, may mitigate the pathological impact of *BAAT* loss, as also observed in other genetic cholestasis models [17, 35]. Combining *BAAT* deficiency with *Cyp2c70* KD or knockout may therefore yield a more human‐like BA profile and a sensitized model for dissecting the pathophysiology of *BAAT* deficiency.

Our AAV8‐mediated *Baat* KD model has practical advantages. It enables adult‐onset, liver‐directed suppression of *BAAT* expression in mice, thereby avoiding developmental confounders inherent to germline knockout models. The reproducibility of the biochemical phenotype and its compatibility with integrated isotope‐tracing assays make it a powerful platform for dissecting BA synthesis and regulation. Nevertheless, the model also has limitations. KD efficiency varies between individuals, and residual *BAAT* activity may partially mask downstream effects. Moreover, the present analyses were performed at a single time point, 2 weeks after vector administration, and thus capture early metabolic responses rather than long‐term adaptations. Conditional or chronic knockout models that allow sustained, controllable *BAAT* suppression will enable longitudinal assessment of metabolic adaptation and potential disease progression.

Clinically, the concordant elevation of 7‐HOCA in both patient and mouse highlights this metabolite as a candidate biomarker of *BAAT* deficiency or related disorders of BA synthesis. Longitudinal studies correlating circulating 7‐HOCA with hepatic or systemic outcomes will be essential to validate this potential. Beyond its diagnostic utility, the identification of enhanced cholesterol oxidation links conjugation failure and dysregulated BA biosynthesis. This insight broadens the conceptual framework of *BAAT* deficiency from a terminal conjugation defect to a disorder of systemic BA homeostasis, suggesting the need to evaluate metabolic intermediates in patients with unexplained cholestasis or BA synthesis defects.

In conclusion, *BAAT* deficiency is associated with persistent elevation of 7‐HOCA and coordinated changes in BA synthesis and tissue distribution. Evidence supports activation of the classical pathway, with a possible contribution from the acidic branch, alongside impaired biliary export. Our *Baat* KD model recapitulates these features and provides a tractable platform for mechanistic and therapeutic studies.

## Author Contributions

Soma Koga performed the animal experiments, analyzed and interpreted the results, and drafted the manuscript. Hajime Takei performed the bile acid analysis and contributed to data interpretation. Ryutaro Tamura established the experimental conditions for the animal studies and contributed to data interpretation. Yugo Takaki collected the clinical information and biological samples of the *BAAT*‐deficient patient and contributed to the interpretation of the related experiments. Hiroyuki Kusuhara revised the manuscript. Hiroshi Nittono conceived the study, contributed to the design of the patient‐related study and to the interpretation of the results, and revised the manuscript. Hisamitsu Hayashi conceived and supervised the study, contributed to the design and interpretation of all experiments, and drafted and revised the manuscript. All authors approved the final version of the manuscript.

## Funding

This work was supported by Japan Agency for Medical Research and Development (AMED) grant JP25ek0109679 and JP25ek0109818, JSPS KAKENHI Grant Number JP24KK0144, Kawano Masanori Memorial Public Interest Incorporated Foundation for Promotion of Pediatrics Grant Number 36–17, Japan Research Foundation for Clinical Pharmacology, and the Kobayashi Foundation to Hisamitsu Hayashi. The funding source did not participate in the study design and execution.

## Ethics Statement

The study involving human subjects was approved by the institutional review board of the Graduate School and Faculty of Medicine, Kyoto University (approval number: G1253). This study was conducted as part of the nationwide registry study CIRCLe (Comprehensive and Informative Registry system for Childhood Liver disease), in which genetic and bile acid analyses are routinely performed for all enrolled patients. The study adhered to the principles of the Declaration of Helsinki (1964) and its subsequent amendments, or to comparable ethical standards. All mouse experiments were approved by the animal experiment committee of the University of Tokyo (permission number: A2024P003–01), conducted in accordance with the institutional guidelines, and reported in accordance with the ARRIVE guidelines.

## Consent

Informed consent was obtained primarily through electronic signatures via a secure online system approved by the ethics committees, and in some cases, written consent was obtained. Consent was obtained from all participants or from their parents/guardians for minors under 18 years of age.

## Conflicts of Interest

The authors declare no conflicts of interest.

## Supporting information


**Table S1:** Reagent used in this study.
**Table S2:** sgRNA sequences used in this study.
**Table S3:** Plating medium used in this study.
**Table S4:** QPCR primer sequences used in this study.

## Data Availability

The data supporting the findings of this study are not publicly available due to ethical and regulatory restrictions, including patient confidentiality and institutional governance of registry‐based research, but are available from the corresponding author upon reasonable request.
